# 

**Published:** 1905-09

**Authors:** 


### Society Notes.

THE SEVENTH COUNCILOR DISTRICT MEDICAL SOCIETY, formerly the Austin District Medical Society, will meet in Austin, at 10 a. m., September 21st inst. A large attendance is expected.

BRAZOS VALLEY MEDICAL ASSOCIATION.—The twentieth semi-annual meeting of the Brazos Valley Medical Association will be held at Rockdale, Texas, on the second Tuesday and Wednesday in November, the 14th and 15th days thereof. You are earnestly requested to prepare a paper on a theme of your own selection. You have ample time. Please do not delay in this matter, which is of such vital importance to our grand order. We need your aid now more than at any other time. Send the the title of your paper to Dr. W. B. Briggs at Easterly, at once, so the programs may be out early. You are very urgently requested to attend this meeting, as business of the gravest importance will be transacted. Please do not delay answering. Write at once.

MISSISSIPPI VALLEY MEDICAL ASSOCIATION.—At the next meeting of the Mississippi Valley Medical Association, to be held at Indianapolis, Ind., October 10th, 11th and 12th, the annual addresses will be delivered by Dr. Arthur R. Edwards, of Chicago, and Dr. W. D. Haggard, of Nashville, Tenn. Dr. Edwards has chosen for the subject of his address "Certain Phases of Uremia, Their Diagnosis and Treatment," and Dr. Haggard will discuss in his address "The Present Status of Surgery of the Stomach." In addition to these addresses there will be the annual address of the President, Dr. Bransford Lewis, of St. Louis. A cordial invitation is extended to every physician in the valley to attend this meeting, for which a large number of interesting and valuable papers have been promised.

TRI-STATE (ALABAMA, GEORGIA AND TENNESSEE) MEETING.—The seventeenth annual meeting of the Tri-State Medical Society of Alabama, Georgia and Tennessee will be held in Chattanooga, Tuesday, Wednesday and Thursday, September 26, 27 and 28, 1905. A rate of one fare for the round trip has been secured on account of the fall meeting of the Chattanooga Fair Association. This organization will have a horse show and other attractions September 26th to 30th. Membership to this Association is open to all members of the profession in good standing, and a most cordial invitation is extended to all such medical men. A program of unusual merit is already assured, and those who have not yet sent the subject of their papers should do so at once to the Secretary, Dr. Raymond Wallace, Chattanooga, Tennessee.

